# Human Teeth-Derived Bioceramics for Improved Bone Regeneration

**DOI:** 10.3390/nano10122396

**Published:** 2020-11-30

**Authors:** Ki-Taek Lim, Dinesh K. Patel, Sayan Deb Dutta, Han-Wool Choung, Hexiu Jin, Arjak Bhattacharjee, Jong Hoon Chung

**Affiliations:** 1 Department of Biosystems Engineering, Kangwon National University, Chuncheon 24341, Korea; dbhu10@gmail.com (D.K.P.); sayan91dutta@gmail.com (S.D.D.); 2 Department of Oral and Maxillofacial Surgery and Dental Research Institute, School of Dentistry, Seoul National University, Seoul 151921, Korea; woolmania@naver.com; 3 Department of Plastic and Traumatic Surgery, School of Stomatology, Beijing Stomatological Hospital, Capital Medical University, Beijing 100069, China; hyuksoo725@daum.net; 4 Department of Materials Science and Engineering, Indian Institute of Technology, Kanpur 208016, India; arjak.ceramic16@gmail.com; 5 Department of Biosystems and Biomaterials Science and Engineering, Seoul National University, Seoul 151921, Korea

**Keywords:** human tooth powder, bioceramics, biocompatibility, bone regeneration, vascularization

## Abstract

Hydroxyapatite (HAp, Ca_10_(PO_4_)_6_(OH)_2_) is one of the most promising candidates of the calcium phosphate family, suitable for bone tissue regeneration due to its structural similarities with human hard tissues. However, the requirements of high purity and the non-availability of adequate synthetic techniques limit the application of synthetic HAp in bone tissue engineering. Herein, we developed and evaluated the bone regeneration potential of human teeth-derived bioceramics in mice′s defective skulls. The developed bioceramics were analyzed by X-ray diffraction (XRD), Fourier-transform infrared (FTIR) spectroscopy, and scanning electron microscopy (FE-SEM). The developed bioceramics exhibited the characteristic peaks of HAp in FTIR and XRD patterns. The inductively coupled plasma mass spectrometry (ICP-MS) technique was applied to determine the Ca/P molar ratio in the developed bioceramics, and it was 1.67. Cytotoxicity of the simulated body fluid (SBF)-soaked bioceramics was evaluated by WST-1 assay in the presence of human alveolar bone marrow stem cells (hABMSCs). No adverse effects were observed in the presence of the developed bioceramics, indicating their biocompatibility. The cells adequately adhered to the bioceramics-treated media. Enhanced bone regeneration occurred in the presence of the developed bioceramics in the defected skulls of mice, and this potential was profoundly affected by the size of the developed bioceramics. The bioceramics-treated mice groups exhibited greater vascularization compared to control. Therefore, the developed bioceramics have the potential to be used as biomaterials for bone regeneration application.

## 1. Introduction

Total hip replacement surgery is a massive burden globally due to its tremendous negative impact on the socioeconomic scenario. The total number of hip replacement surgeries in the United States is anticipated to increase more than 1.5 times (~174%) by 2030 [1]. These alarming statistics might force the healthcare community to develop an advanced hospital management model for many patients in a limited space [2] and develop alternate implant material for effective hip replacement surgery [3,4]. Various kinds of bone grafts are utilized to reconstruct bone defects caused by disease or trauma [5]. Autografts are considered a “gold standard” for the bone replacement application. The distinct advantage of histocompatibility without disease transfer risks makes the autografts ideal for bone repair [5,6]. However, their limited availability forces researchers to develop alternative and adequate bone substitutes. Black phosphorus (BP) consists of a single phosphorus element, a homolog to the inorganic constituents of natural bone, and can be applied to treat bone defects [7]. Calcium phosphate-based bioceramics, such as hydroxyapatite (HAp), *β*-tricalcium phosphate, etc., are considered suitable bone graft materials due to their structural and chemical similarity to human bone [8]. These materials are also used as a bioactive coating, bone cement, or in drug delivery applications. Bone contains nanometer-sized crystalline calcium phosphate with dimensions of ~5–20 nm width and 60 nm length [9,10]. Various reports are available highlighting the nano-bioceramic potential for bone tissue applications [11,12].

It has been observed that nanocrystalline HAp powders promote osteoblast adhesion, proliferation, and vascularization [13,14,15,16]. The promising features of nanocrystalline HAp, including better biocompatibility, lack of inflammatory, immunity reactions, and improved osteo-integrative potentials, have appealed to the researchers to fabricate nano-structured scaffolds that mimic natural bone properties [17]. Nanomaterials exhibit fascinating and superior mechanical, optical, and electronic properties due to their high surface-to-volume ratio and specific structural properties that do not occur in micro and/or macro-sized analogs [18]. A significant advancement in the fabrication and application of nanomaterials has been achieved. However, nano-calcium phosphate′s practical applications are still far in the future due to the unknown nano-toxicity and lack of a “standardized” approach to monitor nanomaterial toxicity [19,20]. Various methods, like sol–gel synthesis, co-precipitation, mechanochemical synthesis, hydrothermal reaction, and microemulsion technique, have been applied for the synthesis of nanocrystalline HAp. Among these synthesis techniques, the synthesis quantities are significant concerns, and most of them are restricted to synthesis in small amounts [21]. Therefore, it is necessary to develop an alternative method for synthesizing nanomaterials from natural sources, such as mammalian bone, aquatic sources, shell sources, and mineral sources with improved biocompatibility [22].

We previously evaluated the effects of the sintering temperature for the formation of calcium phosphate-based bioceramics from human teeth for tissue engineering applications. The results indicated that the sintering temperature plays a crucial role in the characteristic properties of the developed bioceramics [23]. Herein, we developed the different sizes of bioceramics from human teeth through heat treatment and comparatively evaluated their bone regeneration potential with commercially available Bio-Oss^®^ (Geistlich Pharma AG, Wolhusen, Switzerland), which is often applied as a bone implant. The developed bioceramics exhibited the Ca/P ratio of 1.67, close to the commercially available HAp. The biocompatibility of prepared samples was monitored by WST-1 assay in the presence of human alveolar bone-derived mesenchymal stem cells (hABMSCs). The cells were healthy and properly adhered to the developed bioceramics. Improved bone regeneration was observed in the defected skulls of mice with the prepared bioceramics, and this potential is profoundly affected by the size of the sample. Higher vascularization occurred in bioceramics-treated mice groups than the control, showing the developed material′s potential for enhanced bone regeneration. The vascularization density was high nano-sized bioceramics compared to micro-sized and commercially available Bio-Oss^®^ material. Therefore, the bone regeneration potential of the developed bioceramics can be fine-tuned by taking different particle sizes.

## 2. Methods

This study has four sections; (a) preparation, (b) analysis of the samples, (c) in vitro, and (d) in vivo investigations related to bone regeneration efficiency.

### 2.1. Preparation of Nano-Calcium Phosphate Bioceramic

Human teeth (18–35 years, Numbers-15) were received from the Department of Oral and Maxillofacial Surgery, Dental Hospital, Seoul National University, Republic of Korea. All procedures for teeth handling were performed under the regulation of an experimental protocol permitted by the Institutional Review Board (IRB) of the Dental Hospital, Seoul National University, Seoul, Korea (IRB No. CRI05008), and informed consent was obtained from each donor. Herein, we performed the 15 donors′ pool to eliminate the donor-specific variability. The received teeth were washed with distilled water to remove the soft tissues from the surface and dried at 50 °C for 48 h in an air oven. The dried teeth were crushed into powders, and crushed powders were heated in an electric furnace (ST-01045, Daihan Scientific, Seoul, Korea) at 1000 °C with a heating rate of 10 °C/min for 2 h in air atmosphere. The heat-treated samples were pulverized with a miller (A10, IKA-WERKE, Nara, Japan), followed by the separation of the developed bioceramics by a sieve (Sieve/Shaker, Daihan Scientific). The diameter of the developed bioceramics occurred in the range of ~50–500 nm. The obtained bioceramics were treated and scrutinized with a NanoSizer Fine Mill (Deaga Powder Systems Co. Ltd., Seoul, Korea) to obtain the nano-sized bioceramics. The sterilization of the samples was performed in an autoclave, followed by cleaning with ultraviolet treatment. The simulated body fluid (SBF) was initially used to evaluate the biomineralization potential of the developed bioceramics (*n* = 3) for different periods (6 and 12 months) at 37 °C. The chemical compositions of the SBF are given in Table S1**.** The SBF solution was kept under mild conditions of pH and near to physiological temperature.

### 2.2. Phase and Microstructural Characterization of Nano-Calcium Phosphate Bioceramics

The microstructure and particle size of fresh and SBF-soaked bioceramics (*n* = 3) were analyzed by a field emission scanning electron microscope (FE-SEM, SUPRA 55VP, Carl Zeiss, Oberkochen, Germany). For this, the sample (fresh and SBF-soaked) powders were coated by a BAL-TEC SCD005 sputter coater for 250 s at 15 mA. The compositional analysis of the fresh and SBF-soaked bioceramics was performed by using energy-dispersive X-ray (EDX) spectroscopy at 30.0 kV. The phase analysis of the fresh bioceramics was accomplished by X-ray diffraction (XRD) (Bruker D5005 X-ray Diffractometer, Kassel, Germany) at room temperature using Cu Kα as the radiation source with a scan speed of 1°/min in the range of 10–90° and an angular range (2θ) (generator was 40 kV, 40 mA and λ (radiation) was 1.5406). The X-ray fluorescence (XRF) spectroscopy (Bruker S4 Pioneer, Karlsruhe, Germany) was used to determine the Ca/P ratio in fresh bioceramics and commercially available HAp using a Rh X-ray source and helium atmosphere. The Fourier-transform infrared (FTIR) spectroscopy (Nicolet 6700, Thermo Scientific, Madison, WI, USA) was performed to determine the functional groups present in fresh and SBF-soaked bioceramics in the range of 400–4000 cm^−1^. The thermal stability of the fresh bioceramics was evaluated through thermal gravimetric analysis (TGA) (SDT Q600, TA Instruments, New Castle, DE, USA) with a 10°/min heating rate from room temperature to 1400 °C.

### 2.3. Cell Culture and Maintenance

The hABMSCs were received from the Korean Cell Line Bank (KCLB; Seoul National University, Korea), and cell culture was performed according to the method described by Gronthos et al. [24]. Briefly, the cells were cultured in α-minimum essential media (MEM) containing 10% fetal bovine serum (FBS, Welgene Inc., Gyeongsan, Korea), 10 mM ascorbic acid (L-ascorbic acid), 1% antibiotics (Anti-Anti, 100×, Gibco-BRL, Gaithersburg, MD, USA), and sodium bicarbonate (Sigma-Aldrich, St. Louis, MO, USA) at 37 °C in a humidified atmosphere with 5% CO_2_ (Steri-Cycle 370 Incubator, Thermo Fisher Scientific, USA) for desired periods. Passage three hABMSCs were used for in vitro experiments.

### 2.4. Cytotoxicity Evaluation

The indirect cell viability of hABMSCs was evaluated by WST-1 assay (EZ-Cytox Cell Viability Assay Kit, Daeillab Service Co. Ltd., Seoul, Korea) in the presence of the developed bioceramics as reported earlier in somewhere else [25]. In brief, the extraction media were prepared by soaking the samples in a serum-free media (SFM), and incubated for 24 h. The cells (1 × 10^4^) were seeded in 96-well plates in SFM and incubated at 37 °C and 5% CO_2_ environment for 24 h. After this, the media were changed with the extracted media and further incubated for 24 h. The media without any extract were considered as control. After 24 h of incubation, the cultured cells were washed with PBS and treated with 10 µL of WST-1 dye and further incubated for 2 h. The formed formazan concentration was measured with a spectrophotometer (Victor 3, Perkin Elmer) at 450 nm (reference wavelength 625 nm). All experiments were performed in triplicate (*n* = 3, size ~60 nm), and values are expressed as mean ± standard deviation (SD).

### 2.5. Cell Morphology

The adhesion behavior of hABMSCs in the presence of the prepared bioceramics was evaluated through FE-SEM after 1 and 7 days of the treatment. Briefly, the cells were seeded in a 35 × 10 mm culture disc in the presence of the developed bioceramics (*n* = 3, size ~60 nm) and incubated at 37 °C and 5% CO_2_ conditions. The cultured cells were washed with PBS and fixed with 4% paraformaldehyde (PFA) (Sigma-Aldrich, USA) for 1 h at 4 °C. The fixed cells were washed with PBS and treated with ethanol. The samples were sputter-coated with platinum, and images were captured by an FE-SEM.

### 2.6. Animal Care, In Vivo Bone Formation and Vascularization Study

The imprinting control region (ICR) (male, 32–34 g, six weeks old) mice were purchased from Orient Bio, Gapyeong-gun, Korea. All mice were kept in an insulated and soundproof room at an ambient temperature of 21 ± 2 °C, with a constant relative humidity of 35 ± 2% with an automatically controlled 12 h light and 12 h dark cycle (lights off at 20:00 h). Sufficient amounts of food and water were supplied to the mice for their care. The experimental mice were divided into two groups (Group = 2, and total mice = 6). The one group represented the negative (without any treatment) and positive control (with commercially available Bio-Oss^®^, size 0.25–1 mm, Geistlich Pharmaceutical, Wolhusen, Switzerland), and the other group showed the experimental conditions with different sizes of bioceramics. The in vivo study was performed as reported in an earlier work [23]. Briefly, the mice were treated with ether, followed by the intraperitoneal supplement of 0.3–0.4 mL of 20 mg/mL Avertin (Sigma-Aldrich, USA) for anesthetization. The 5% iodine solution was used to disinfect the mice skull. After this, a 1–1.5 cm sagittal incision was created very carefully on the scalp with a sterile surgical blade, followed by creating a critical defect (~5 mm diameter) in the central area of the calvaria bone. The pockets were cleaned with surgical gauze, filled with commercially available Bio-Oss^®^, and different bioceramics sizes for four weeks. The pocket with no sample was considered as a negative control. The soft tissue was repositioned and sutured carefully after the implantation of the materials. After the four weeks of treatment, the animals were sacrificed with isoflurane, and each sample was recovered and treated with 4% formalin (paraformaldehyde in 1× sterile PBS). No leaching was observed from one implant site to the neighboring site after four weeks of treatment. For histological analysis, hematoxylin and eosin (H&E) staining was performed. Decalcification of the newly developed calcified tissue was done with a 10% aqueous formic acid solution. The decalcified section images were taken by a light microscope (BX-50, Olympus Optical Co., Tokyo, Japan). All experiments were performed under the guideline of the approved animal protocol.

### 2.7. Micro-Computed Tomography (µCT) Analysis

The µCT was performed to analyze the prepared bioceramics in vivo bone regeneration efficiency, as previously described [26]. Briefly, the treated skull was excised, cleaned, and fixed by 4% PFA, and visualized by a µCT analyzer (Spectra Lago X, Tucson, AZ, USA) at 20 µm resolution. The images were captured, and the new bone formation was examined.

### 2.8. Statistical Analysis

Statistical analysis was carried with one-way ANOVA using the Origin Pro 9.0 software (Origin Pro v9.0, Origin Lab Corp., Northampton, MA, USA). All experiments were performed in triplicate (*n* = 3), and the results are expressed as mean ± standard deviation (SD). Statistical significance was considered as * *p* < 0.05.

## 3. Results

**Figure 1:**
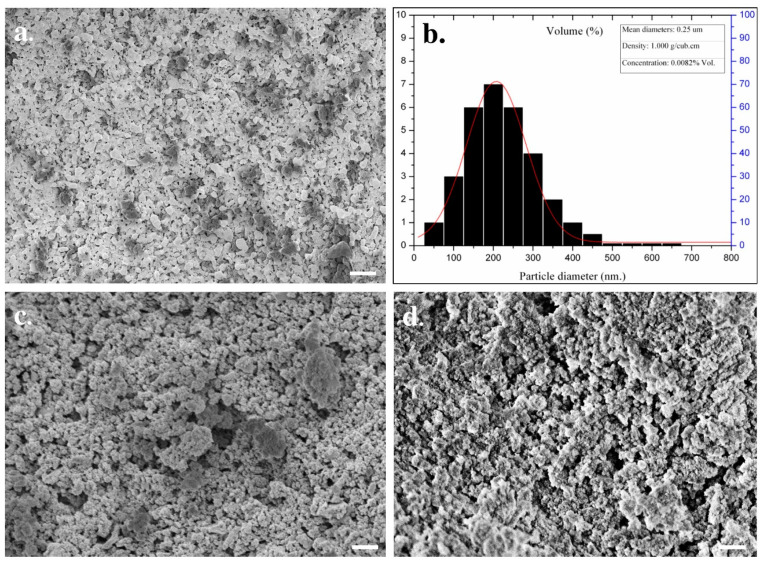
Surface morphology of human teeth-derived bioceramics through heat treatment; (**a**) the FE-SEM image of fresh sample; (**b**) particle size distribution curve of fresh bioceramics (*n* = 3, 50 particles); (**c**) the FE-SEM image of SBF-soaked bioceramics (6 months); and (**d**) the FE-SEM image of SBF-soaked bioceramics (12 months) (Scale bar = 10 μm).

**Figure 2:**
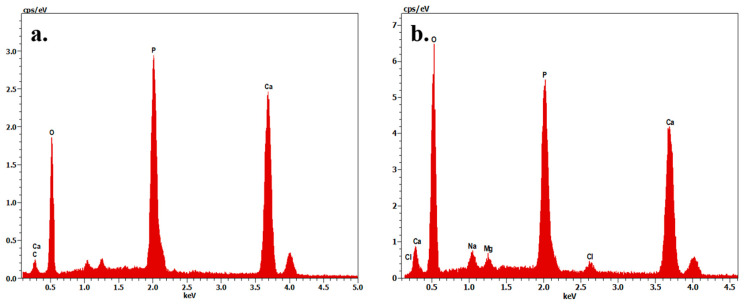
The energy-dispersive X-ray (EDX) profile of the prepared bioceramics; (**a**) fresh; and (**b**) SBF-soaked bioceramics (12 months).

**Figure 3:**
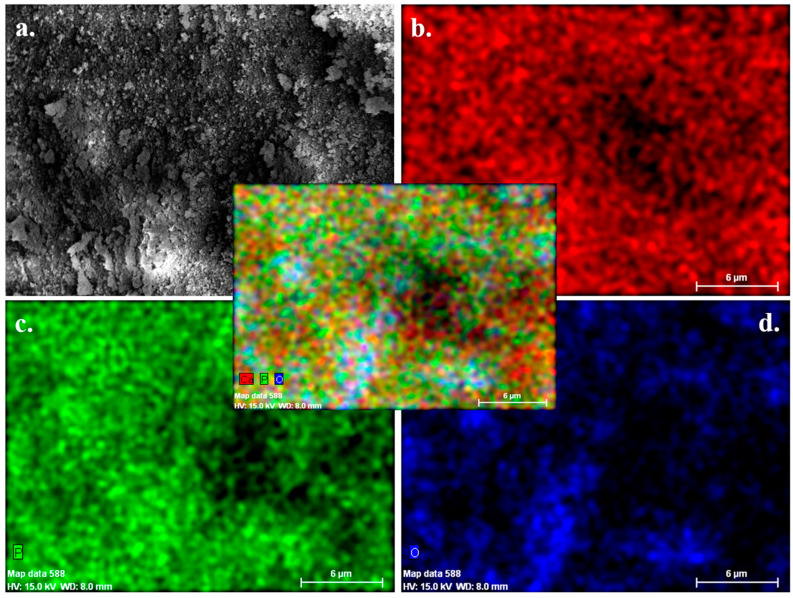
(**a**) The EDX elemental mapping of human teeth-derived bioceramics; (**b**) calcium; (**c**) phosphorous; and (**d**) oxygen (a merged image is shown at the center).

The microstructural characteristics of human teeth-derived bioceramics were examined through FE-SEM measurement, and the morphologies are presented in Figure 1a. The particle diameter of the prepared bioceramics occurred in the range of 50–500 nm (Figure 1b). The FE-SEM morphologies of SBF-soaked bioceramics after different time intervals are presented in Figure 1c,d. The SBF-soaked bioceramics exhibited the deposition of the granular particles on their surface. The elemental composition of commercially available HAp and prepared bioceramics was determined by X-ray fluorescence (XRF) spectroscopy, and the results are given in Table 1. It was noted that the Ca/P molar ratio was 1.67. Teeth powders contain some low-level metal impurities (i.e., Na, Mg, Al, K, Zn, Fe, Cu), which may be attributed to traces supplied by the chemical reagents or biomass trace elements. The EDX spectra of fresh and SBF-soaked bioceramics are shown in Figure 2a,b. The EDX result indicated that the bioceramics were mainly composed of calcium (Ca), phosphorus (Pa), and oxygen (O). The trace amounts of other elements, such as magnesium and chlorine, were also noted in the SBF-soaked bioceramics. The elemental analysis of fresh and 12-month SBF-soaked bioceramics through energy-dispersive X-ray (EDX) spectroscopy is given in Table 2. The EDX elemental mapping of human teeth-derived bioceramics is given in Figure 3. The obtained results indicated that the O, P, and Ca were the main constituents of the developed bioceramics, and they were uniformly distributed in the sample.

**Figure 4:**
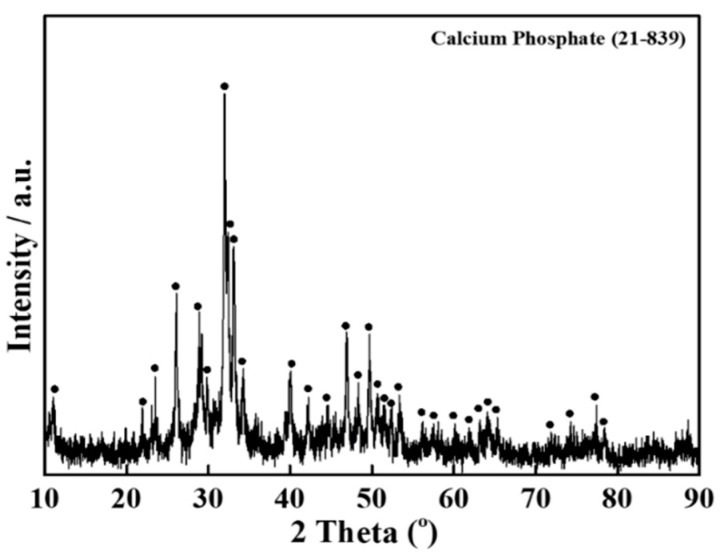
The XRD pattern of human teeth-derived bioceramics through heat treatment.

**Figure 5:**
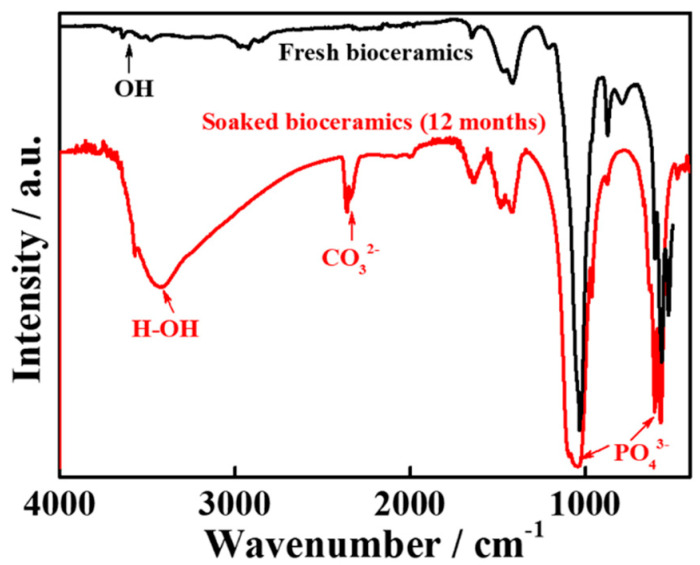
The FTIR spectra of fresh and SBF-soaked (12 months) bioceramics.

**Figure 6:**
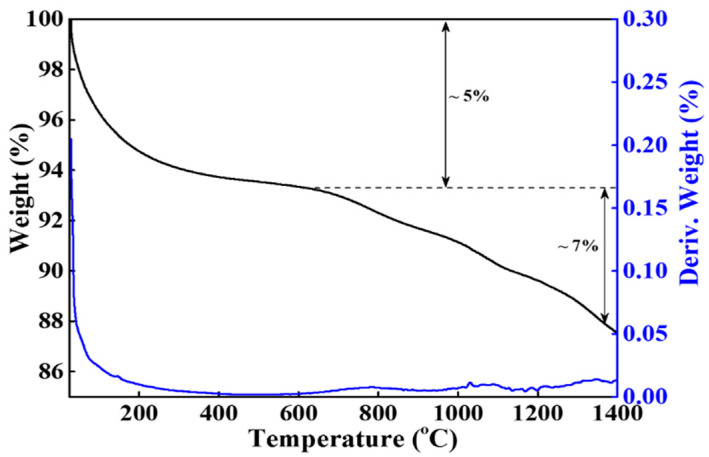
Thermal stability of human teeth-derived bioceramics; black line for TGA curve and blue line shows their derivative curve for fresh bioceramics under inert condition (N_2_ atmosphere).

The XRD pattern of human teeth-derived bioceramics is shown in Figure 4. The XRD pattern resembled the previously reported pattern of HAp, indicating that heat treatment facilitates the formation of natural HAp from human teeth [27]. The XRD peak at 2θ = 33.3° suggested the existence of the CaP moiety in developed bioceramics [28]. The presence of the different functional groups in bioceramics was examined through the FTIR spectroscopy. The FTIR spectra of fresh and SBF-soaked (12 months) bioceramics are shown in Figure 5. The FTIR results are identical to the HAp pattern [27]. The appearance of several peaks in the regions of 3532, 2530–2329, 1640–1620, and 960–570 cm^−1^ matched the corresponding absorption peaks of HAp. The appearance of these peaks indicated the presence of different ions such as phosphate (PO_4_^3−^), hydroxyl (OH^−^), and carbonate (CO_3_^2−^) in bioceramics. The thermal stability of the developed bioceramics was determined through TGA, and the obtained thermogram is given in Figure 6. The TGA curve shows the ~5% weight loss in the lower temperature region (up to 200 °C). A small endothermic transition was observed in this region in the DTA curve, but no noticeable weight loss was noted in the range of ~400 °C to 600 °C. However, a continuous weight loss was observed above 600 °C. The total weight loss of the bioceramics was ~12% at 1400 °C.

**Figure 7:**
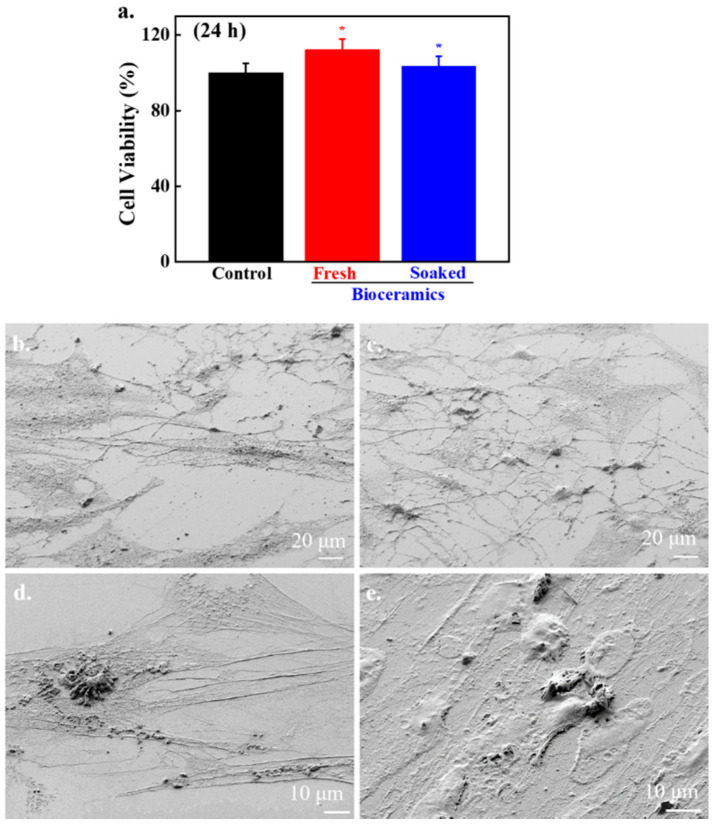
Cytotoxicity evaluation of human teeth-derived bioceramics; (**a**) indirect cell viability data after 24 h of treatment with the extracted media of fresh and SBF-soaked bioceramics (12 months); (**b**,**c**) the FE-SEM morphologies of the cultured cells in the presence of fresh bioceramics after 1 and 7 days of treatment at low magnification (1000×); and (**d**,**e**) high magnification (3000×), respectively.

**Figure 8:**
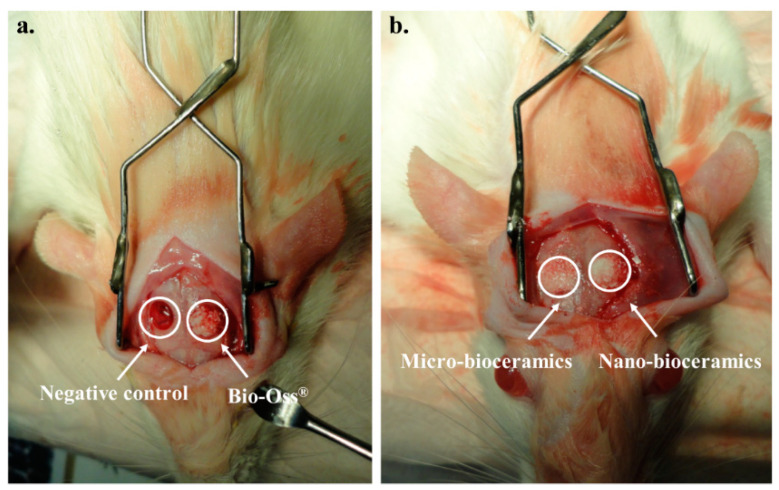
Images of in vivo transplantation; (**a**) with negative and positive control; and (**b**) in the presence of different sizes of bioceramics.

**Figure 9:**
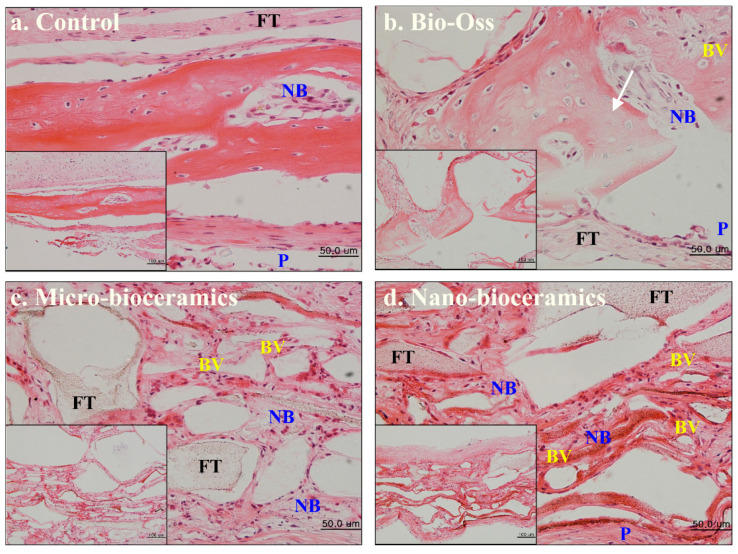
Hematoxylin and Eosin (H&E)-stained images of human-teeth derived bioceramics; (**a**) negative control; (**b**) positive control; (**c**) with micro and nano-sized bioceramics; and (**d**) in the presence of nano-sized bioceramics after four weeks of transplantation (Scale bar: 50 and 100 μm) Here, NB, P, BV, and FT indicate the new bone, periosteum, blood vessel, and fibrous tissue, respectively.

**Figure 10:**
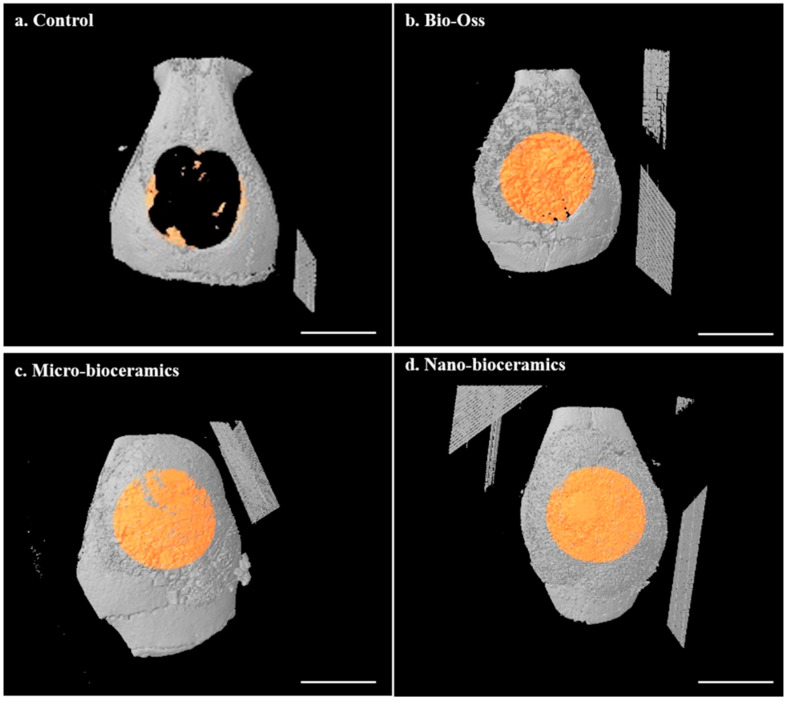
Micro-computed tomography (µCT) images of the defected mice skull in the presence of indicated materials after four weeks of transplantation; (**a**) control, (**b**) Bio-Oss, (**c**) micro-bioceramics, and (**d**) nano-bioceramics (Scale bar: 2 mm). The percentage bone healing was ~7 ± 2.08, 92 ± 3.27, 88 ± 4.70, and 95 ± 2.14% for the control, Bio-Oss, micro, and nano-bioceramics, respectively.

Cytotoxicity of fresh and SBF-soaked bioceramics (6 months) was monitored by WST-1 assay in the presence of hABMSCs after 24 h of incubation, and the results are given in Figure 7a. No adverse effects were observed on hABMSCs in the presence of fresh and SBF-soaked bioceramics, indicating their biocompatibility. The FE-SEM morphologies of the hABMSCs cultured on developed bioceramics at different periods are shown in Figure 7b,c. The cultured cells were healthy and proliferated under the experimental treatment, showing their biocompatibility. Bone regeneration potential of the developed bioceramics was evaluated through in vivo transplantation experiment using six-week-old imprinting control region (ICR) male mice. The groups without any treatment and with commercially available Bio-Oss^®^ were considered as negative and positive control. The bioceramics (micro and nano-sized) treated groups were taken as experimental groups. The images of in vivo transplantation are shown in Figure 8a,b. No inflammation was observed around the transplanted area after four weeks of treatment, suggesting that the developed bioceramics were nontoxic and biocompatible. Rapid bone regeneration occurred in the presence of developed bioceramics, and this efficiency was intensely affected by the size of the developed bioceramics. The H&E staining process was performed to assess the histological analysis of the conducted experiment after four weeks of transplantation, and the results are given in Figure 9. The formation of new connective tissues and capillaries was observed around the transplanted area after four weeks of treatment, and its density was high in bioceramics-treated groups compared to the control and commercially available Bio-Oss^®^ (Figure 9c,d). Various real-time non-invasive imaging techniques were applied to detect the bone-healing or correct placement during the implantation. These were X-ray computed tomography (CT), magnetic resonance imaging (MRI), and ultrasound (US), which can monitor the natural repair and the fate of host–materials interaction, in vivo [29]. CT images of the defected male mice skulls under different experimental conditions are shown in Figure 10. This work was different from our previous work, where we had evaluated the effects of sintering temperature on the formation of calcium-based bioceramics for tissue engineering [23].

## 4. Discussion

Bone graft materials should have osteo-inductive potential to achieve optimal bone regeneration results. Their selection depends on clinical considerations and patient morbidity. The autogenous bone graft is considered the gold standard in the graft materials due to its inheritance osteo-inductive property. However, it has several disadvantages, including graft material availability, increased grafting time, blood loss, and additional cost [3]. The allogenic bone graft has better availability than autograft and does not require the second surgical procedure, but there is a chance of disease transmission and adverse immune responses, limiting its broad applicability [3]. Allogenic bone grafts are considered to be superiorly osteoconductive but weakly osteo-inductive and non-osteogenic [30]. Allogenic bone grafts can be obtained from living donors (femoral head), multiple organ donors, and post mortem donors (bone). The most important step in reducing immunogenicity and disease transmission is fluid pressurization to eliminate bone marrow and cellular debris. The detailed medical, social, sexual, and behavioral history of allograft donors is performed to further detect disease transmission risk factors. The number of the intraoral donor site depends on the quantity, geometry, and type of the bone required for reconstruction [31].

Therefore, we need an alternative and safe substitute for bone grafts. Synthetic bioceramics can be applied as a bone grafts substitute, but its limited availability and toxicity are serious concerns. Hence, finding an alternate source of biocompatible ceramics will increase their application in the tissue engineering sector. Herein, the bioceramics developed from human teeth were used as a bone reconstructing material in vivo. The fresh bioceramics demonstrated a particle-like morphology, while a rough surface and partially porous structure were in SBF-soaked bioceramics. The sintering kinetics played an important role in the size of the bioceramics. It was observed that the pore size and crystal morphology of material obtained from human and bovine bone samples were profoundly affected by the calcination temperature [32]. The bioactivity studies in SBF showed granular particle morphology due to the calcification of the samples in the media, and the ball-like apatite layer was also observed in the FE-SEM images, which proved the bioactivity of the samples. The elemental mapping analysis enabled the information related to the uniform distribution of the elements and the inhomogeneity in fresh and SBF-soaked bioceramics [33]. The appearance of the sharp peaks in the XRD pattern showed the crystalline nature of the developed bioceramics, which was extensively influenced by the sintering temperature. An enhancement in the crystallinity was reported earlier in bovine bone-derived HAp by increasing the calcination temperature 600 °C to 1000 °C [34]. A decrease in the crystallinity occurred above 1000 °C in Hap, owing to the decomposition of HAp into β-TCP [35,36]. Therefore, 1000 °C is assumed as a suitable calcination temperature for the formation of crystalline HAp. The Ca/P ratio in the developed bioceramics was determined by XRF analysis, and it was 1.67. This value is close to the commercially available HAp (1.61) [37]. SBF-soaked bioceramics exhibited a broad FTIR absorption peak of –OH compared to fresh bioceramics due to the presence of adsorbed moisture moiety [38]. As evidenced in the TGA curve, the weight loss was due to the removal of moisture moiety from the sample [39], further supported by a small endothermic transition in this region in the DTA curve. The continuous weight loss from the samples above 600 °C can be attributed to the dehydroxylation of HAp and its conversion to whitlockite [40]. No exothermic peaks occurred in the developed bioceramics, indicating the sample′s high purity [41].

Fresh and SBF-soaked bioceramics had no adverse effects on hABMSCs, indicating their biocompatibility. It is well-known that cellular activity is significantly affected by material properties such as surface roughness, texture, and surface chemistry [42,43]. They can change the cell′s proliferation by changing the physico-chemical interactions between the material surface and cells through ionic forces [44]. Cell adhesion potential offers the information associated with the biomaterial′s possible use as an implant [43]. The FE-SEM images indicated that the cells were healthy and adhered adequately to flattened osteoblast-like morphology connected by the filopodia. The cell density was increased with increasing the culture time. In vivo study demonstrated that the bone regeneration efficiency was high in bioceramics-treated groups compared to the control, which was more significant in nano-sized bioceramics. This was probably due to their superior biocompatibility. The size effects of hydroxyapatite nanoparticles on human osteoblast-like MG-63 cells were also studied by Shi et al. They observed that the hydroxyapatite with the smallest diameter exhibited superior cellular activity in their macro-analogs [45]. It has been noted that nano-sized HAp incorporated composites mimicked natural bone conditions and supported better growth and proliferation of human osteoblast cells [46]. Nano-sized HAps are widely used in the field of tissue engineering for the rapid regeneration of bone tissues. However, selecting a suitable material for these applications still poses a challenging task for the researcher [47]. The osteogenic potential of calcium phosphate (CaP)-based bioceramics was earlier reported, and this potential is profoundly affected by the bone-like apatite layer formation and the absorption of protein on the surface of the materials [48,49]. The CaP-based bioceramics are considered bioactive materials and have excellent biocompatibility and osteo-conductivity potential. The phase composition and porosity extensively influence the osteo-conductivity of the materials.

HAp and other CaP-based bioceramics can regulate cell differentiation via the osteogenic process and promote bone tissue regeneration. Enhanced vascularization was observed in bioceramics-treated groups and their density further increased in nano-sized bioceramics-treated mice. Micro CT and histological analysis suggested that the defected mice skulls were nearly filled with new tissues in the presence of developed bioceramics compared to the control. Following the X-ray images, the micro and nano-sized bioceramics-treated groups showed better integration with the native bone after four weeks of transplantation (Figure 10). The HAp has a porous structure, facilitating the infiltration and migration of osteoblast cells from host bone to the defected sites [50,51,52]. No donor-to-donor variability issues were observed in the bioceramics developed to use as an implanted biomaterial. This study was limited to evaluating the bone regeneration potential of bioceramics derived from waste human teeth. However, more detailed clinical studies are required to assess any adverse effects of the developed bioceramics to allow their commercial applications. The limitations of biological-derived ceramic bone grafts are their batch variability, which can be minimized by the pooling of the donor sources [53]. It has been observed that the donor-specific variabilities such as cell proliferation, cell viability, and osteogenic differentiation occurred in human mesenchymal stromal cells (MSCs) obtained from different donors, which restricted their wide applicability. Several factors, like donor′s age, sex, disease, and hormone status, are responsible for the donor-specific variability. Therefore, the pooling of human MSCs from different donors is applied to minimize the donor-specific variabilities. This approach has already been utilized in the past and can be used in bone tissue engineering [54]. Hence, our work demonstrates that heat treatment facilitates the formation of a useful bioactive material from waste human teeth for the rapid regeneration of bone tissues, and derived nano-sized bioceramics can be considered a suitable material for tissue engineering.

## 5. Conclusions

This study investigated the preparation and characterization of calcium phosphate-based bioceramics from human teeth. The FTIR spectra indicated the presence of characteristic peaks of the HAp, which was also confirmed through the XRD pattern. The appearance of sharp diffraction peaks in XRD suggested their crystallinity. The Ca/P molar ratio was determined through EDX and XRF techniques, and it was 1.60 and 1.67, respectively, which is closed to the commercially available hydroxyapatite (1.61). An enhancement in the Ca/P molar ratio (1.80) was observed in the SBF-soaked sample rather than in the fresh due to the calcification in SBF conditions. The particle size of the prepared bioceramics occurred in the range of approximately 50–500 nm diameter. No adverse effects were noted on hABMSCs in the presence of developed bioceramics, indicating their biocompatibility. The cells were healthy and appropriately adhered to the developed bioceramics.

Additionally, improved bone tissue regeneration occurred in the presence of prepared bioceramics rather than in the control, and commercially available Bio-Oss^®^ demonstrated their superior osteogenic potential. This potential is significantly affected by the size of the bioceramics. Based on these findings, we concluded that nano-sized calcium phosphate-based bioceramics have great potential for bone tissue application.

## Figures and Tables

**Table 1:** The determination of percentage element composition for commercially available HAp and human teeth-derived bioceramics through X-ray fluorescence (XRF) spectroscopy and their Ca/P molar ratio.

Product	Ca_5_(PO_4_)_3_(OH)	Bioceramics
Ca	39.9	36.66
P	18.5	16.40
Ca/P Ratio	1.61	1.67
Na	-	0.79
Mg	-	0.60
Al	-	0.04
Si	-	0.07
Cu	-	0.01
Cl	-	0.20
K	-	0.03
Zn	-	0.14
Sr	-	0.03
Y	-	0.04
Zr	-	0.60
Ag	-	0.02
Fe	-	0.01
S	-	0.03

**Table 2:** Elemental analysis of human-teeth derived bioceramics under different conditions through energy-dispersive X-ray (EDX) spectroscopy.

	Composition (%)	
Element	Fresh Bioceramics	SBF-Soaked Bioceramics (12 Months)
Calcium	35.70	40.50
Phosphorus	16.70	16.66
Oxygen	47.60	39.91
Sodium	-	1.23
Chlorine	-	1.09
Magnesium	-	0.61
Total	100.00	100.00

## Supplementary Materials

The following are available online at https://www.mdpi.com/2079-4991/10/12/2396/s1, Table S1: The chemical compositions of the SBF solution used in this study.
