# Supplementary material for: Human Teeth-Derived Bioceramics for Improved Bone Regeneration
Source: Nanomaterials (Basel). 2020 Nov 30;10(12):2396. doi: 10.3390/nano10122396 (PMC7761315; doi:10.3390/nano10122396)
Supplement: Supplementary file 1 [file nanomaterials-10-02396-s001.pdf]

Supplementary Materials

Human Teeth-Derived Bioceramics for Improved Bone Regeneration

Ki-Taek Lim [1,*,†], Dinesh K. Patel [1,†], Sayan Deb Dutta [1], Han-Wool Choung [2], Hexiu Jin [3], Arjak Bhattacharjee [4] and Jong Hoon Chung [5,*]

[1] Department of Biosystems Engineering, Kangwon National University, Chuncheon 24341, Korea; dbhu10@gmail.com (D.K.P.); sayan91dutta@gmail.com (S.D.D.)

[2] Department of Oral and Maxillofacial Surgery and Dental Research Institute, School of Dentistry, Seoul National University, Seoul 151921, Korea; woolmania@naver.com

[3] Department of Plastic and Traumatic Surgery, School of Stomatology, Beijing Stomatological Hospital, Capital Medical University, Beijing 100069, China; hyuksoo725@daum.net

[4] Department of Materials Science and Engineering, Indian Institute of Technology, Kanpur 208016, India; arjak.ceramic16@gmail.com

[5] Department of Biosystems and Biomaterials Science and Engineering, Seoul National University, Seoul 151921, Korea

† These authors contributed equally to the paper.

* Correspondence: ktlim@kangwon.ac.kr (K.-T.L.); jchung@snu.ac.kr (J.H.C.)

Table S1. The chemical compositions of the SBF solution used in this study.

Reagent	Amount (g/L)
NaCl	6.547
$NaHCO_3$	2.268
KCl	0.373
$Na_2HPO_4 \cdot 2H_2O$	0.178
$MgCl_2 \cdot 6H_2O$	0.305
$CaCl_2 \cdot 2H_2O$	0.368
Na_2SO_4	0.071
$(CH_3OH)_3CNH_2$	6.057
